# The first rare case of *Candida palmioleophila* infection reported in China and its genomic evolution in a human host environment

**DOI:** 10.3389/fmicb.2023.1165721

**Published:** 2023-07-26

**Authors:** Na Wu, Yusheng Wu, Yunzhuo Chu, Zhihui Ren, Hailong Li, Chen Rong, Min Yang, Ning Jiang, Yanyan Jiang, Jingjing Chen, Jingping Zhang, Sufei Tian

**Affiliations:** ^1^Department of Infectious Diseases, The First Hospital of China Medical University, Shenyang, China; ^2^National Clinical Research Center for Laboratory Medicine, Department of Laboratory Medicine, The First Hospital of China Medical University, Shenyang, China; ^3^Department of Intensive Care Unit, Shenyang Fourth People’s Hospital of China Medical University, Shenyang, China

**Keywords:** *Candida palmioleophila*, yeast, whole-genome sequence, *ERG11*, ITS

## Abstract

**Introduction:**

*Candida palmioleophila* is a rare human pathogenic fungus, which has been poorly characterized at the genome level. In this study, we reported the first fatal case of *C. palmioleophila* infection in China and investigate the microevolution of *C. palmioleophila* in the human host environment.

**Methods:**

A series of *C. palmioleophila* stains were collected from the patient at different time points for routine microbial and drug sensitivity testing. The first *C. palmioleophila* isolate 07202534 was identified by *de novo* whole genome sequencing. The *in vitro* and *in vivo* genetic evolutionary characteristics of *C. palmioleophila* were discussed based on the analysis of bioinformatics data.

**Results:**

The six *C. palmioleophila* isolates displayed dose-dependent sensitivity to fluconazole. The *C. palmioleophila* genome contained homologous genes such as *CDR1* and *MDR1,* which were recognized to be related to azole resistance. In addition, amino acid variation was detected at F105L and other important sites of *ERG11*. In addition, the mean divergence time between *C. palmioleophila* and *Scheffersomyces stipites* CBS 6054 was 406.04 million years, indicating that *C. palmioleophila* originated earlier than its closest relative. In addition, the six strains of *C. palmioleophila* isolated form the patient had higher homology and fewer mutation sites, which indicated the stability in *C. palmioleophila* genome. We also found that *C. palmioleophila* had a wide natural niche and may evolve slowly.

**Discussion:**

We believe that this study will contribute to improve our understanding of the genetic evolution, pathogenicity, and drug resistance of *C. palmioleophila* and will aid in the prevention and control of its spread.

## Introduction

*Candida palmioleophila* is ascomycetous yeast, which was first isolated in 1988 from soil. In 1999, *C. palmioleophila* was reported as a human pathogen causing intravenous catheter-associated fungemia (Sugita et al., 1999). In recent years, the incidence of *C. palmioleophila* infections has risen, and is often associated with drug resistance (Datta et al., 2015; Mroczynska and Brillowska-Dabrowska, 2019; Casagrande Pierantoni et al., 2020; Lavergne et al., 2023). In 2018, *C. palmioleophila* was isolated for the first time in China and showed resistance to fluconazole (Xiao et al., 2018). But there was no clinical case described in previous report. Here, we reported the first fatal case of *C. palmioleophila* infection in China.

In clinical practice*, C. palmioleophila* is often misdiagnosed as *Candida famata* (*Debaryomyces hansenii*) (Desnos-Ollivier et al., 2008) or *Candida guilliermondii* (*Meyerozyma guilliermondii*) (Jensen and Arendrup, 2011). Sequence analysis of 18S, ITS1, 5.8S, ITS2, and D1/D2 has shown that *C. palmioleophila* is closely related to *C. famata* and *C. guilliermondii* (Stavrou et al., 2019). At presence, little known about the genetic relationship between *C. palmioleophila* and *C. famata* or *C. guilliermondii,* as well as common pathogenic fungi. Although the draft genome sequence of *C. palmioleophila* has been published in 2023 (Lavergne et al., 2023), the genetic evolutionary characteristics of *C. palmioleophila* are still lacking. In addition, the pathogenic genes and drug resistant genes of *C. palmioleophila* need to be fully explored at the genomic level. To get a more comprehensive genome, using the hybrid approach (Illumina Novaseq + Pacbio) perform *de novo* whole genome sequencing (WGS) of *C. palmioleophila* and compare its genetic and evolutionary characteristics with those of related species.

## Materials and methods

### Clinical information and ethics statement

Clinical information (e.g., age, sex, presence of underlying diseases, and antifungal drug usage) relating to the *C. palmioleophila*-positive patient was collected via the Hospital Information System (HIS) system of the First Hospital of China Medical University.

This study was reviewed and approved by the Ethics Review Committee (ERC) of the First Hospital of China Medical University (ERC number: 2019-53-2) and was conducted in accordance with the Declaration of Helsinki and its amendments.

### Description of the first fatal case of *Candida palmioleophila* infection in China

A 92-year-old man with aspiration pneumonia was admitted to the First Hospital of China Medical University on March 31, 2020. The patient’s chief complaint was fever for 4 days. He had acute onset of fever and respiratory symptoms. The patient was admitted for routine blood tests. His white cell count was 7.87 × 10^9^ cells/L; neutrophil accounted for 85%; and a C-reactive protein level, 22.6 mg/L. Lung computed tomography (CT) indicated multiple spots under both lungs. Because aspiration pneumonia was suspected, the patient was administered antibiotics such as cefotaxime or meropenem. Aspiration pneumonia recurred several times.

The patient also had a 10-year history of Alzheimer’s disease, a 5-year history of cerebral infarction, and had been bedridden for a long time with nasogastric feeding. In addition, he had a history of prostatic hyperplasia for many years, a long-term indwelling catheter, and bullous pemphigus for 3 months. Several skin ulcerations could be seen on the patient’s limbs and trunk.

On June 9, 2020, a white floc was found in the patient’s indwelling catheter, which did not obstruct catheter drainage. The catheter was replaced and the bladder was rinsed with normal saline. The midstream clean-catch urine cultures were negative for bacteria, but the first *C. palmioleophila* strain (isolate 07202534) was isolated. The serum (1, 3)-β-D-glucan level was 227.86 pg./mL. Voriconazole 200 mg (twice daily; which was double the initial dose) was administered by nasal feeding.

The patient developed gastrointestinal bleeding on July 16, 2020. Diet and water were prohibited while parenteral, nutritional support was given via peripheral inserted central catheter (PICC).

On July 28, 2020, the patient developed a fever and was in a poor condition. Urinalysis showed elevated leukocyte numbers [87.38–632.38 cells/high power field (HPF)]. *C. palmioleophila* was isolated several times from a mid-stream urine sample (Table 1). Because disseminated fungal infections were not excluded, this patient had been treated with caspofungin (an initial dose of 70 mg, followed by a daily dose of 50 mg) for 15 days (between July 28, 2020 and August 3, 2020). However, even the patient was received the new therapy, his urine cultures stayed positive for *C. palmioleophila*.

**Table 1:** Characterization and susceptibility testing of the six *Candida palmioleophila* isolates.

Date	Isolate	Source	Colony count cfu/mL	MIC (μg/mL)						
				FLU	IZ	VOR	AMB	5-FC	CAS	MF
2020-06-09	07202534	Urine	1×10 ~ 5	32	1	1	1	<0.06	0.06	0.12
2020-07-13	08201515	Urine	1×10 ~ 6	32	2	1	1	<0.06	0.06	0.12
2020-07-27	08201684	Urine	1×10 ~ 4	32	4	2	1	<0.06	0.06	0.06
2020-08-06	08201782	Urine	1×10 ~ 5	32	2	1	1	<0.06	0.06	0.12
2020-08-18	07204494	Urine	1×10 ~ 5	32	4	1	1	<0.06	0.12	0.06
2020-10-12	07206383	Catheter	200	64	1	1	1	<0.06	0.06	0.06

Note: AMB, amphotericin B; 5-FC, flucytosine; FLU, fluconazole; IZ, itraconazole; VOR, voriconazole; CAS, caspofungin; MF, micafungin.

On October 12, 2020, *C. palmioleophila* was isolated from the patient’s PICC catheter tip. The infection progressed, and the patient died of septic shock on October 16, 2020.

### Clinical strain isolation

On June 9, 2020, a urine culture yielded a *Candida* sp. (isolate 07202534), with a count of 1 × 10^5^ colony-forming units (CFU)/mL. This strain could not be identified using the VITEK 2, MALDI-TOF, or VITEK-MS systems, and was later confirmed as *C. palmioleophila* by internal transcribed spacer (ITS) sequencing (Table 1). C. *palmioleophila* strains were detected in the patient’s urine samples on July 13 (isolate 08201515), July 27 (isolate 08201684), August 6 (isolate 08201782), and August 18 (isolate 07204494); the colony counts ranged between 10^4^ and 10^6^ CFU/mL. On October 12, 2020, a strain of *C. palmioleophila* (isolate 07206383) was isolated from an intravenous catheter tip, with a colony count of 200 CFU/mL (Table 1).

*In vitro* antifungal susceptibility testing of these isolates was performed using the Sensititre YeastOne colorimetric microdilution method (Thermo Fisher Scientific, Waltham, MA, United States). The minimum inhibitory concentration (MIC) results were interpreted according to the breakpoints suggested for other *Candida* species by the Clinical Laboratory Standards Institute (CLSI; reference M27-S4).

### Whole genome resequencing

Whole genome resequencing (WGS) was performed by Shanghai Personal Biotechnology Co., Ltd. (China). *C. palmioleophila* cell were cultured on YPD medium at 37°C for 18 h and then used for genomic DNA extraction. The genomic DNA was extracted using the cetyltrimethylammonium bromide (CTAB) method, with minor modifications (Huang et al., 2018). The DNA concentration, quality, and integrity were determined using a Qubit Flurometer (Invitrogen, United States) and a NanoDrop Spectrophotometer (Thermo Scientific, United States). *De novo* sequencing was performed on the first *C. palmioleophila* isolate (07202534). Paired-end libraries were prepared using the TruSeq DNA Sample Preparation Kit, followed by 2 × 150 bp sequencing using the Illumina Novaseq platform (both from Illumina, United States). Then 10-kb libraries were generated with the Template Prep Kit (Pacific Biosciences, United States). Single-molecule real-time (SMRT) sequencing was performed by using the Pacific Biosciences platform (Pacific Biosciences, United States). Raw data were processed after adapter contamination removal and data filtering using the Adapter Removal (Schubert et al., 2016) and SOAPec tools (Luo et al., 2012). The filtered reads were assembled using SPAdes (Bankevich et al., 2012) and A5-miseq (Coil et al., 2015) to construct scaffolds and contigs. The Flye software was used to assemble the data obtained by Pacbio platform sequencing (Kolmogorov et al., 2019). Subsequently, all assembled results were integrated to generate a complete DNA sequence. Finally, the *C. palmioleophila* genome sequence was acquired after rectification with Pilon software (Walker et al., 2014).

### Prediction and annotation of function genomic elements

Function genomic element prediction involved the prediction of coding genes, non-coding RNA, and repeated sequence. Genes prediction was performed using Augustus (version 3.03), glimmerHMM (version 3.0.1), and SNAP (version 2006-07-28). Homologous gene prediction was obtained from the protein sequences of related species using the “exonerate” tool (version 2.2.0). The predicted genes were integrated using EVidenceModeler software (version r2012-06-25) (Ter-Hovhannisyan et al., 2008). The completeness of the assembled genome was also evaluated using BUSCO software (version 5.1.2), with comparison to the fungi_odb10 lineage dataset (creation date: September 10, 2020; number of BUSCO markers: 758) (Manni et al., 2021a,b). After obtaining the assembly genome, tandem repeats and interspersed repeats were identified by using RepeatModeler and RepeatMasker software (Tempel, 2012). The RepBase database was used to predict sequences that were similar to known repeat sequences. Structure prediction was performed using RepeatModeler software. RepeatMasker was used to make the prediction using the constructed repeat sequence library. In the analysis of non-coding RNA, tRNA was predicted by tRNAscan-SE (Lowe and Eddy, 1997), rRNA was predicted by RNAmmer1.2 (Lagesen et al., 2007), and the prediction of other non-coding RNAs was obtained by comparing with Rfam (Griffiths-Jones et al., 2005). Protein-coding genes were predicted by Augustus (version 3.03) (Stanke and Morgenstern, 2005), glimmerHMM (version 3.0.1) (Majoros et al., 2004), GeneMark-ES (version 4.35) (Ter-Hovhannisyan et al., 2008), and exonerate (version 2.2.0),^1^ and the prediction results were integrated by EVidenceModeler (version r2012-06-25) (Haas et al., 2008).

Functional annotation was completed by performing a Basic Local Alignment Search Tool (BLAST) search of the following databases: including Non-Redundant (NR) protein database, Gene Ontology (GO), Kyoto Encyclopedia of Gene and Genomes (KEGG), SwissProt, Evolutionary Genealogy of Genes: Non-supervised Orthologous Groups (EggNOg), Pathogen-Host Interactions (PHI), Transporter Classification Database (TCDB), Carbohydrate-Active Enzymes (CAZy), cytochrome P450, and Pfam.

### Genomic variation analysis

For the other five *C. palmioleophila* isolates, the extracted DNA of was randomly interrupted by ultrasound, and an insert fragment with an average length of about 400 bp was obtained according to different interrupted time and rotation speed, and then the fragment was constructed for subsequent genomic library. It is typically 6–8 cycles at the time of library construction. The sequencing data were then mapped to the above genome sequence of the *C. palmioleophila* isolate (07202534) to identify single nucleotide polymorphisms (SNPs), gene acquisition, recombination, loss as well as nucleotide insertions and deletions (indels). The filtered high quality data were aligned to the reference genome using the bwa-men program (version 0.7.12-r1039); the aligned parameters were based on the default parameters of bwa-men. The sam files were transformed to bam files using the bwa sampe and samtools (0.1.19-44,428 cd) toolkit. Picard software (version 1.107)^2^ was used to sort the bam files and remove duplicates using the “MarkDuplicates” tool. The Realigner Target Creator command in the GATK^3^ package was then used to output a file containing all possible indels; the reads were also realigned around all the indels to improve the accuracy of mutation prediction.

### Comparative genome analysis

#### Gene family analysis

First, the protein sequence of the reference genome (Supplementary Table S5) was downloaded, screened according to the length of the protein sequence, and the sequence with sequence length less than 50 amino acids was removed. All the protein sequences to be analyzed constituted a dataset. At the same time, the data set was used as a query for All-*vs*-all blastp analysis, and the threshold of series alignment was set as 1e-10. The orthomcl (version 2.0.8) software was used to process the result of sequence alignment, in which the length of sequence alignment was set to 70%, MCL was used to cluster the gene family, and I (Inflation) used for clustering was set to 1.5. Finally, a homemade Perl script is used to sort out and count the clustering results.

#### Single-copy orthologous gene phylogenetic tree

Based on the results of the homologous gene cluster analysis, single copy homologous genes were selected for multiple sequence alignment. An alignment quality control software system (MAFFT software was used for sequence alignment^4^ and Gblocks software was used for quality control)^5^ was used to remove unreliable sequence alignment sites. The ML algorithm, the model of amino acid in the FastTree software is used to construct phylogenetic tree.^6^ The reliability of the phylogenetic tree branches needs to be verified after constructing the evolutionary tree. Sequence alignment was visualized using the itoL website.

#### Time analysis of divergent evolution

The mcmctree program (Bayesian method) of Paml software was used to estimate the divergence time between species based on amino acid sequence alignment information (Yang, 2007). According to the TimeTree website^7^ recorded divergence time for calibration: infer the divergence time of each variety (branch is confidence interval).

#### The expansion and contraction of gene families

The expansion and contraction of gene families was analyzed using CAFÉ software (version 3.1), which used statistical analysis to obtain the size of each gene family. For a given phylogenetic tree, CAFÉ can estimate the total birth and death rates of each gene family, infer the most likely gene family size at each internal node, and identify branches where the gene family expands and contracts if the gene family size is known for each existing species. NCBI nr (Database release 2017.10.10), KEGG (KAAS Ver. 2.1) and GO annotation (InterPro version 66.0, release 2017.11.23) were performed on genes related to expansion and contraction of the target gene family.

## Results

### Microbiological characteristics of clinically isolated *Candida palmioleophila*

**Figure 1:**
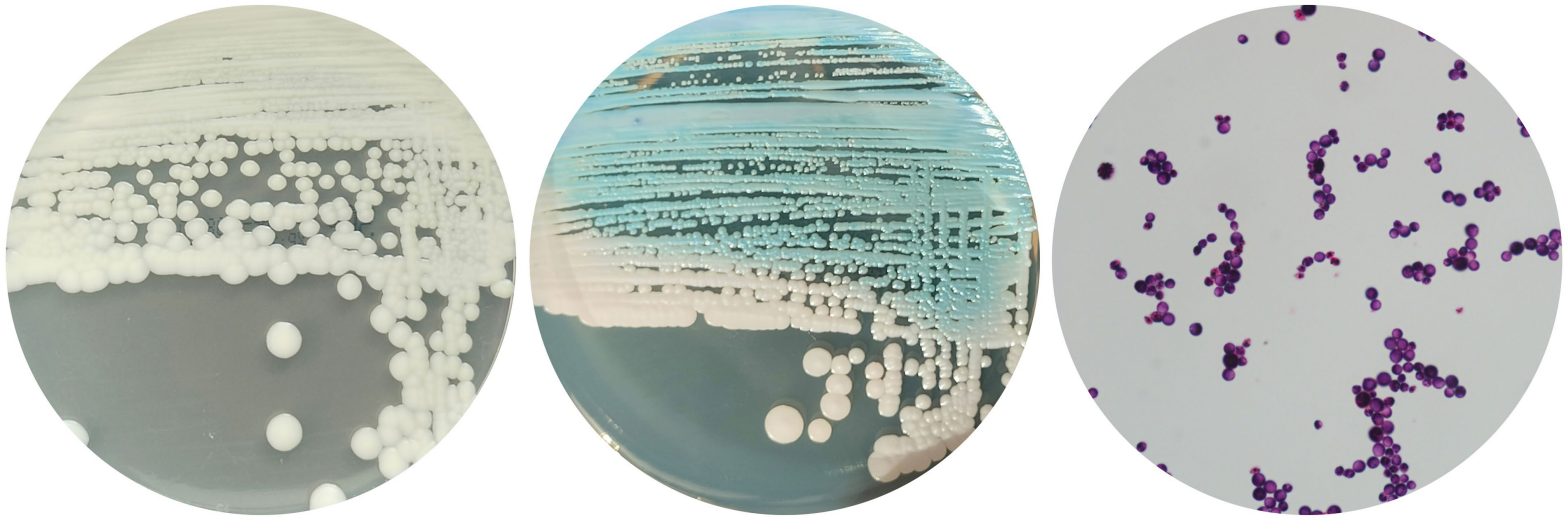
Colonies of *Candida palmioleophila* grown on Sabouraud dextrose agar at 35°C for 3 days **(A)**; or CHROMagar at 35°C for 3 days **(B)**; **(C)** A microscopy image of *C. palmioleophila* (×1,000).

On Sabouraud dextrose agar, *C. palmioleophila* grew as round, smooth, white, creamy colonies with neat edges at 35°C for 3 days. When grown on CHROM agar *Candida* medium (CHROMagar, Paris, France), the colonies appeared white at 35°C for 24 h, gradually turning turquoise, with some areas of light pink at 35°C for 5 days. *C. palmioleophila* can be grown at temperatures up to 40°C. *C. palmioleophila* did not form pseudohyphae, but budding was observed under the microscope (Figures 1A–C).

All six *C. palmioleophila* isolates were sensitive to fluconazole in a dose-dependent manner and were susceptible to 5-fluorocytosine, itraconazole, voriconazole, amphotericin B, caspofungin, and micafungin (Table 1).

### *De novo* genome sequencing and assembly of the first *Candida palmioleophila* isolate (07202534)

To decipher the molecular characteristics of *C. palmioleophila*, *de novo* genome sequencing was performed, and the genome was assembled. The basic features of the *C. palmioleophila* genome were as follows: a total of 29,906,210 reads containing 4,485,931,500 bases was obtained by Illumina Novaseq sequencing; the GC content was 40.06%; and 97.68, and 93.16% for the Q20 and Q30 quality scores, respectively. After data filtering, 29,686,002 high-quality reads were obtained, accounting for 99.26% of the total Reads. The Pacbio Sequel system sequenced 3,222,909 reads containing 34,695,115,316 bases; the GC content was 40.17% and the N50 was 11,528 bp. The sequencing data were assembled using splicing software, and 10 scaffolds and 10 contigs were obtained. The N50 of all the scaffolds was 2,004,991 bp. The BUSCO integrity of the assembled genome was 97.7%. Additional details, such as the comparison of *C.-palmioleophila*-associated genomic information with that obtained in another study (Lavergne et al., 2023), are provided in Table 2.

**Table 2:** Genomic assembly and functional annotation of the *Candida palmioleophila* genome.

Item	Value		Item	Count	Percentage (%)
	This study	Other study^*^			
Total length (bp)	12,762,530	12,588,764	NR	4,343	98.41
Max length (bp)	2,999,745		EggNOG	4,213	95.47
GC content (%)	40.17	39.96	KEGG	2,978	67.48
Gene number	4,413	5,332	SwissProt	3,968	89.92
Total gene number (bp)	6,821,815		GO	3,605	81.69
Gene/Genome (%)	53.45		P450	4,314	97.76
Contigs	10		TCDB	980	22.21
Scaffolds	10	196	Pfam	3,779	85.63
Contigs N50	2,004,991		PHI	1, 249	28.30
Scaffolds N50	2,004,991	2,030,000			
Contigs N90	658,531				
Scaffolds N90	658,531	550,000			

^*^Other study by Lavergne et al. (2023).

### Annotation of the *Candida palmioleophila* isolate 07202534 genome

**Figure 2:**
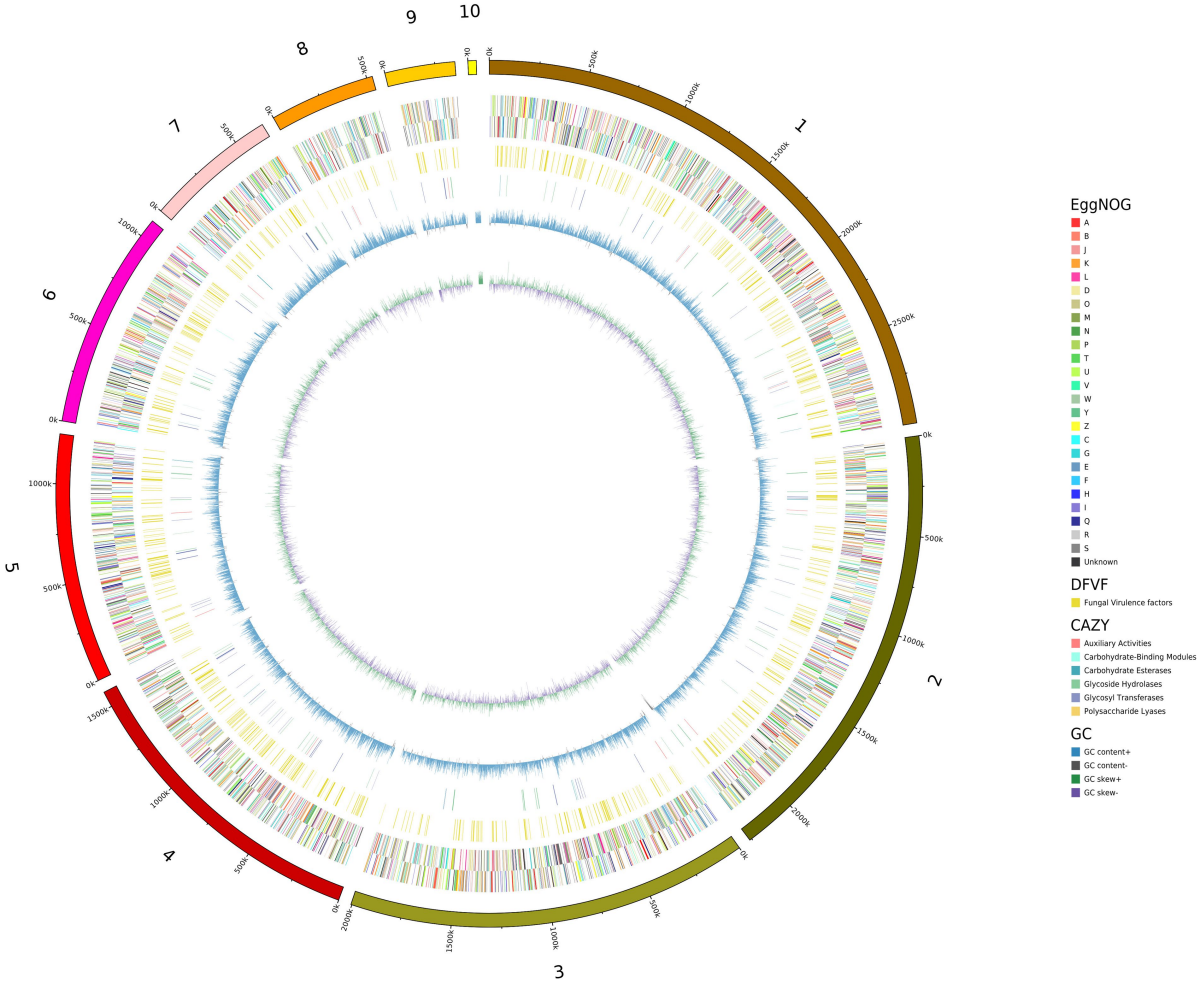
A circle map of the *Candida palmioleophila* genome. The map was drawn using circos-0.69-6, according to the results of gene prediction and annotation analyzes. Inside to outside: First lap: GC skew; Second circle: G + C content; Lap 3: CAZy; Fourth lap: DFVF; Fifth circle: COG to which each CDS on the negative sense chain belongs; Circle 6: COG to which each CDS on the justice chain belongs; Seventh circle: The scale.

We used *de novo* and homologous prediction to annotate the *C. palmioleophila* genome. In total, we identified 4,413 coding genes (comprising 6,821,815 bp), which accounted for 53.45% of the genome length; each gene had an average of 1 exon. Protein coding genes were annotated using the NR, EggNOg, KEGG, SwissProt, GO, P450, TCDB, Pfam, and PHI databases and yielded 4,343 (98.41%), 4,213 (95.47%), 2,978 (67.48%), 3,968 (89.92%), 3,605 (81.69%), 4,314 (97.76%), 980 (22.21%), 3,779 (85.63%), and 1, 249 (28.30%) genes, respectively (Table 2 and Figure 2), Detailed Functional annotations of *C. palmioleophila* gene encoding proteins using the EggNOg, KEGG, GO, PHI, and CAZy database were shown in Supplementary Figures S1–S5.

### Using the SwissProt database to evaluate the conservation of *Candida palmioleophila* genes involved in azole resistance and pathogenesis

We next annotated the *C. palmioleophila* genome using the SwissProt database and found that *C. palmioleophila* contained conserved gene components of the ergosterol biosynthesis pathway (e.g., *ERG11*), which is associated with azole resistance. Moreover, the similarity between the *ERG6* gene of *C. palmioleophila* and *Candida albicans* was 89.5%. In addition, we found genes encoding the ATP binding cassette (ABC) transporter family *CDR1* and major facilitator superfamily (MFS) transporter *MDR1* in *C. palmioleophila* genome. However, the identity between these *C. palmioleophila* genes and their orthologs in related species such as *Saccharomyces cerevisiae, C. albicans,* and *C. tropicalis* was not overly high (33.7–89.5%).

Many gene families involved in the pathogenesis in *C. albicans,* such as the secreted lipases (*LIP1*) and secreted aspartyl proteinases (*SAP1, SAP 9, SAP 30, SAP 61, SAP 114, SAP 145, SAP 155, SAP 185,* and *SAPT1*) were also enriched in *C. palmioleophila*. This suggests that *C. palmioleophila* may display similar pathogenesis as *C. albicans* (Supplementary Table S1).

**Figure 3:**
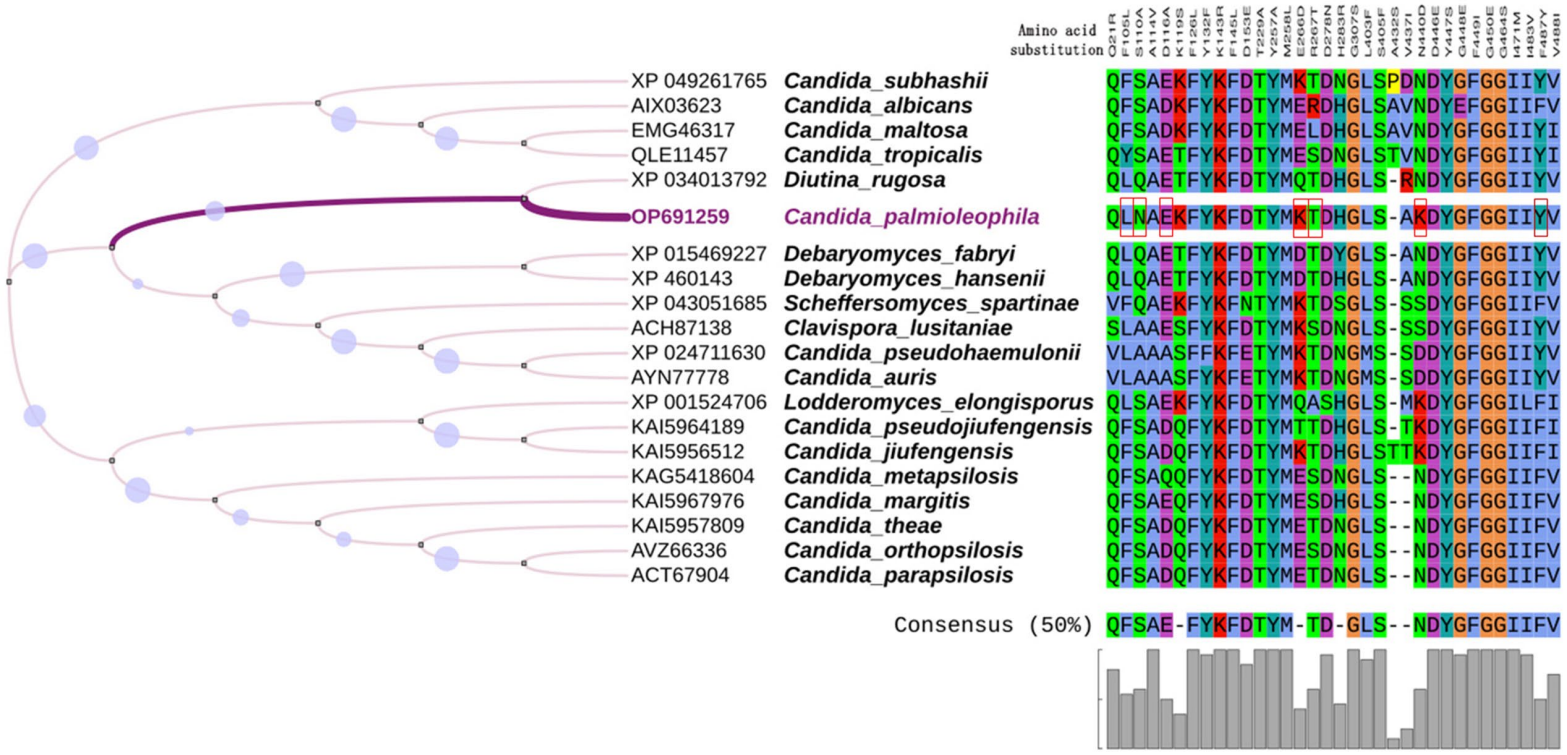
Multiple alignment of ERG11. The ERG11 amino acid sequences of 20 common *Candida* species were analyzed, focusing on amino acid mutation sites related to fluconazole resistance. Red boxes indicate the amino acids substitutions in *C. palmioleophila* that are associated azole resistance. Amino acid numbers are based on the *C. albicans* protein sequence. MEGA7.0 was used for sequence comparison and the evolutionary tree was generated using iTOL software.

*ERG11* encodes lanosterol 14 α-demethylase, which is targeted by fluconazole. The *C. palmioleophila ERG11* shared 72.5% of its sequence identity with the *C. tropicalis* ortholog (Genbank P14263). Furthermore, we downloaded the *ERG11* sequence of 19 common *Candida* spp. from the NCBI website, combined them with the scaffold2.t669/OP691259 sequence generated in this study, and analyzed the amino acid mutation sites related to fluconazole resistance. By comparing the *C. palmioleophila ERG11* with its homolog in *C. albicans*, we found that the amino acid mutations occurred in important sites such as F105L, S110N, D116E, E266K, R267T, N440K, and F487Y. Among these, the F105L, E266K, and F487Y amino acid mutation sites were consistent with the fluconazole resistance of *C. auris* and other closely related species (Munoz et al., 2018; Figure 3).

### The phylogenetic analysis of 37 *Candida palmioleophila* its sequences

**Figure 4:**
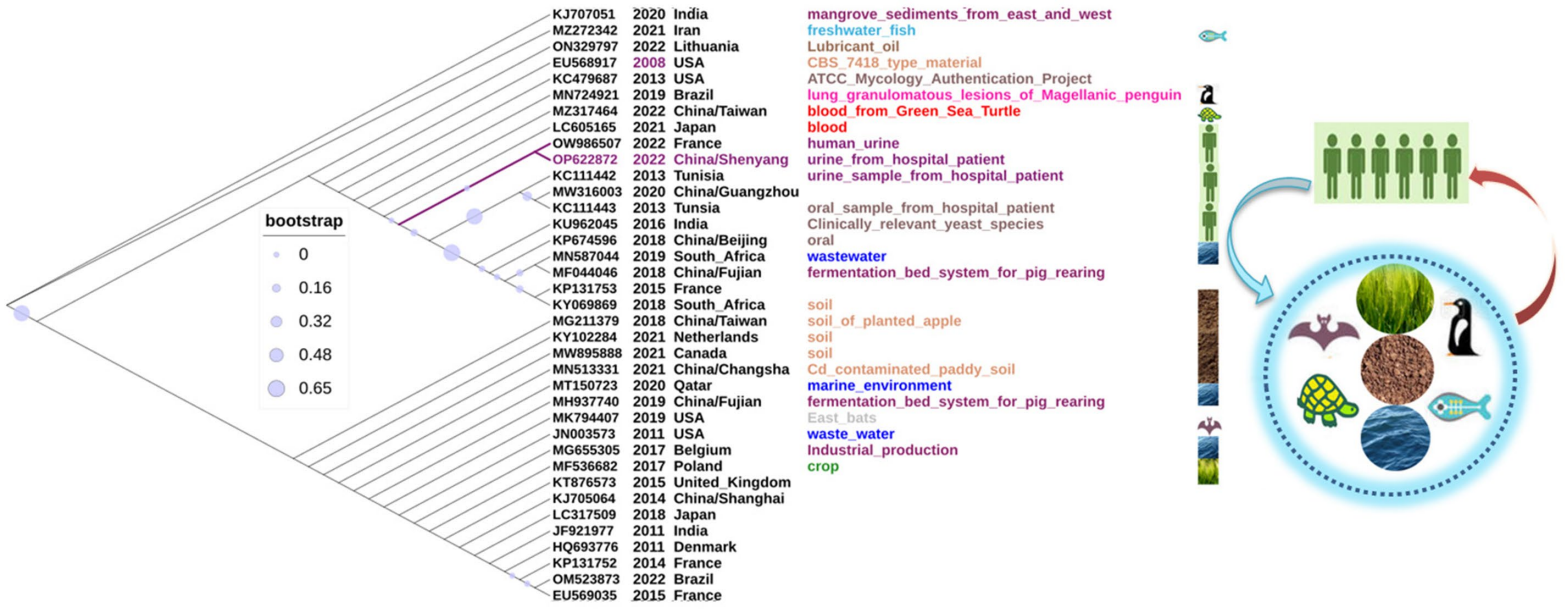
The phylogenetic analysis of 37 *Candida palmioleophila* internal transcribed spacer sequences was performed using MEGA7.0, and the phylogenetic tree was generated using iTOL software.

In all, 37 *C. palmioleophila* ITS sequences, including the dataset OP622872 submitted in this study, were downloaded from the NCBI website. These strains of *C. palmioleophila* were isolated between 2008 and 2022, predominantly from across Asia, Europe, Africa, North America, and South America. These *C. palmioleophila* isolates came from a variety of sources including marine, soil, wastewater, industrial product, crop, animal (turtle, bat, fish, and penguin) and human blood, tissue, urine, and oral specimens. Evolutionary analysis showed that the ITS sequences of these strains were highly homologous (Figure 4). These results suggest that *C. palmioleophila* resides in a wide range of natural environments and can adapt to harsh environments. Changes in the global environment caused by human behavior can lead to changes in the distribution of yeast species, meaning that *C. palmioleophila* could become an emerging human pathogen in the near future.

### Genomic variation analysis of the other five *Candida palmioleophila* isolates

For the series of *C. palmioleophila* strains, we found that the mapping rate was 97.7198.73% and coverage ≥20X was 99.96%, indicating that they were highly homologous. In addition, we found very few SNP, indel, or copy number variation (CNV) sites between these strains, suggested that the genomes of *C. palmioleophila* were highly stable (Supplementary Tables S2–S4).

Using the complete genome of the *C. palmioleophila* isolate 07202534 as the reference genome, we found a total of 42 SNPs in the five subsequent *C. palmioleophila* isolates. Of these, 25 SNPs were different among five *C. palmioleophila* strains. No exon changes were found. However, eight of the SNPs occurred in the upstream region of the *SGT1* gene, and another eight SNPs occurred in the upstream region of the coding gene that encodes the plasma membrane-associated coenzyme Q6 reductase (PGA3) protein. There were also eight SNPs in the upstream region of the gene encoding for the prenylated Rab acceptor 1 (YIP3) protein (Supplementary Table S2).

For next examined indel mutations and found that only one catheter-associated *C. palmioleophila* isolate (07206383) had a 46-bp exonic deletion in the gene encoding for the transcriptional repressor TUP1. The other 51 indels occurred in the upstream, downstream, and intergenic gene regions, including those of *SGT1, ERG7,* and *LIP1* (Supplementary Table S3).

Large region duplication CNVs (8,400–37,100 bp) were observed in the five *C. palmioleophila* strains (Supplementary Table S4).

### Comparative genomic analysis of *Candida palmioleophila* and other related species

#### Gene family analysis and the expansion and contraction of gene families

**Figure 5:**
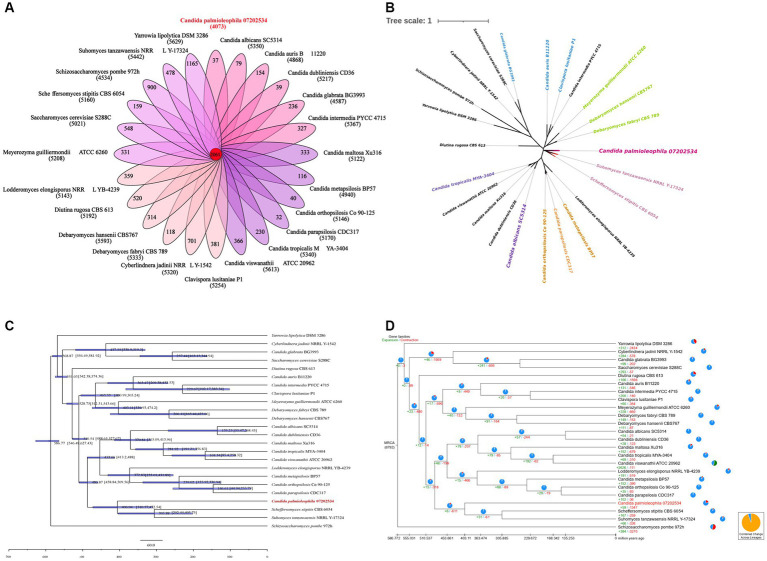
Gene family analysis **(A)**, Single-copy gene evolutionary tree **(B)**, estimation of divergence time **(C)**, and the analysis of the expansion and contraction of gene families **(D)**.

We next performed a gene family analysis of 24 genomes, which included the *C. palmioleophila* isolate 07202534 genome tested in this study and 23 genomes of related fungal species downloaded from the NCBI database (Supplementary Table S5). The results showed that there were 2,061 gene families in the 24 genomes, which included a total of 57,391 genes, among which *C. palmioleophila* isolate 07202534 had 40 unique genes (Figure 5A). According to the results of this analysis, a total of 59 gene families in the target sample expanded and 1,327 gene families contracted over the course of evolution (Figure 5D). Detailed Functional annotations of *C. palmioleophila* expanded and contracted genes encoding proteins using the NR, KEGG, and GO database were shown in Supplementary Figures S6, S7 and Supplementary Tables S6, S7.

#### Single-copy gene evolutionary tree and estimation of divergence time

The phylogenetic tree was constructed based on 388 single-copy genes from 24 fungal genomes. The results showed that the single-copy evolutionary tree could be divided into three main clades. *C. palmioleophila* 07202534, *S. stipitis* CBS 6054, *Suhomyces tanzawaensis* NRRL Y-17324, *C. albicans* SC5314, and eight other species were clustered into one large clade. *C. palmioleophila* 07202534 was closely related to *S. stipitis* CBS 6054 and *S. tanzawaensis* NRRL Y-17324, forming a separate subclade. Because of this close evolutionary relationship, *C. palmioleophila* 07202534 may have some properties similar to those of *S. stipitis* CBS 6054 and *S. tanzawaensis* NRRL Y-17324 (Figure 5B).

The mean divergence time between *C. palmioleophila* 07202534 and the other 11 species in its clade was 486.84 million years (range: 458.84–509.56 million years). The average divergence time between *C. palmioleophila* 07202534 and *S. stipitis* CBS 6054 and *S. Tezawaensis* NRRL Y-17324 was 406.04 million years (range: 310.57–472.54 million years) (Figure 5C).

## Discussion

*C. palmioleophila* belongs to the order Saccharomycetales, the family *Debaryomycetaceae*, and the clade *Candida glaebosa*. *C. palmioleophila* are rare fungi that have been reported to cause an increasing number of human infections (Mroczynska and Brillowska-Dabrowska, 2019; Stavrou et al., 2019; Casagrande Pierantoni et al., 2020; Lavergne et al., 2023). In this study, we reported a rare case of infection with *C. palmioleophila.* To the best of our knowledge, this is the first fatal case of *C. palmioleophila* infection reported in China. An elderly patient developed urinary-tract infection with *C. palmioleophila* for 4 months after the breakdown of the skin barrier (bulbous pemphigus). The patient later developed *Candida* catheter-associated infection and eventually died of septic shock. Two recent studies have shown that *C. palmioleophila* can cause fatal infections in animals. An example was the death of a green sea turtle from disseminated candidiasis and candidemia (Figure 4, MZ317464) caused by *C. palmioleophilla*. The autopsy revealed disseminated *C. palmioleophila* in the joints, bones, brain, and lungs of the animal (Wang et al., 2021). Another case was respiratory candidiasis caused by *C. palmioleophila* in wild Magellanic penguins in Brazil. The autopsy revealed that air sacs and lungs showed marked multifocal to coalescent granulomatous inflammation (Figure 4, MN724921) (Ewbank et al., 2021). These results suggested that *C. palmioleophila* may cause chronic, occult, diffuse lesions, and eventually lead to death in infected individuals. Thus, this rare candida infection should not be underestimated.

There are few studies on the pathogenicity of *C. palmioleophila*. One study suggested that three strains of *C. palmioleophila* had mild-to-moderate virulence (Mroczynska and Brillowska-Dabrowska, 2021). In the present study, the genomic annotation of *C. palmioleophila* using the PHI database suggested that *C. palmioleophila* was only mildly pathogenic (Supplementary Table S4). However, the genome analysis of *C. palmioleophila* 07202534 showed that it was enriched in disease-related genes such as those endocoding secreted lipases (e.g., LIP1) and secreted aspartyl proteinases (SAPs). Surprisingly, we observed a 46-bp indel in the *TUP1* gene in the genome of the catheter-associated *C. palmioleophila* 07205383 isolate. The *TUP1* gene encodes a transcriptional repressor and contributes to the transcriptional control of virulence-associated genes in *C. albicans* (Ruben et al., 2020). Moreover, the similarity between *C. palmioleophila* genes and known homologous pathogenic genes was not high (<50%) (Supplementary Table S1). Thus, the potentially increasing pathogenicity of *C. palmioleophila* and its underlying pathogenic mechanism need to be further studied.

The clinical *C. palmioleophila* isolates identified in this study that exhibited a dose-dependent sensitivity to fluconazole. Besides above mentioned strains, we also isolated several fluconazole-resistant strains from the same patient; however, these strains were not retained and they were not included in this analysis. Thus, these findings collectively suggested that the resistance of *C. palmioleophila* to antifungal medication should not be ignored. Another study confirmed the presence of fluconazole-resistant *C. palmioleophila* infection (Lavergne et al., 2023). In our study, genomic analysis showed that *CDR1, MDR1* and other homologous genes related to azole resistance of clinically isolated *C. palmioleophila.* In addition, we observed amino acids substitutions such as F105L, S110N, D116E, E266K, R267T, N440K, and F487Y in the *ERG11* protein, which were associated with azole resistance. Amino acid variation occurred at important sites such as F105L, E266K and F487Y, which were also targeted by mutations in fluconazole-resistant *C. auris* and *C. lusitaniae* (Munoz et al., 2018). In addition, multiple SNPs occurred in the upstream region of the *SGT1* gene, the product of which acts as a Hsp90 co-chaperone and has been shown to mediate drug resistance in *C. albicans* (Shapiro et al., 2012). The above findings support the inherent resistance of *C. palmioleophila* to fluconazole (Datta et al., 2015). Another study reported that *C. palmioleophila* could develop resistance to echinocandin (Mroczynska and Brillowska-Dabrowska, 2019). Although the *C. palmioleophila* isolates from our patient were sensitive to voriconazole and caspofungin *in vitro*, clinical treatment with these agents was unsuccessful. It was highly likely that the underlying resistance mechanism was not discovered. Therefore, the mechanism of drug resistance should be further studied to reveal the origin of multiple drug resistance in the context of *Candida* infection. The analysis of *C. palmioleophila* genetic characteristics will help to guide clinical and basic research.

In this study, we explored the genetic evolution of *C. palmioleophila* from three aspects:

Firstly, we performed a genome-wide analysis of the *C. palmioleophila* 07202534 isolate, which showed that *C. palmioleophila* 07202534 was closely related to *S. stipites* (which is a CTG[Ser1] yeast) and *S. tanzawaensis* (Figure 5B). The CTG(Ser1) *S. stipitis* has a highly plastic genome (Vega-Estevez et al., 2021), and is found in the gut of wood-eating beetles in hardwood forests or areas with high levels of agricultural waste (Fisher et al., 2018). *C. palmioleophila* can effectively assimilate crude palm oil, and more than six strains of *C. palmioleophila* originate from soil or crop samples (Figure 4). These results suggested that the genetic evolution of *C. palmioleophila* is closely linked to agriculture. In this study, we identified the first case of *C. palmioleophila* in China. We speculated that the *C. palmioleophila* infection was related to the development of agriculture in the region. In addition, at the time of infection, the environment and climatic conditions were favorable for the survival and evolution of *C. palmioleophila.* Moreover, *C. palmioleophila* 07202534 has a distant genetic relationship with the common pathogenic *Candida* spp. such as *C. albicans* and *C. tropicalis*. The genetic distance between *C. palmioleophila*, *C. famata* and *C. guilliermondii*, whose clinical phenotypes are difficult to determine using CHROMagar *Candida* medium, is even greater (Figure 5B), suggesting that *C. palmioleophila* is an emerging fungus causing human infection. We found that the mean differentiation time between *C. palmioleophila* 07202534 and *S. Stipitis* CBS 6054 was 406.04 million years. Meanwhile, the mean differentiation time between *C. palmioleophila* 07202534 and *C. albicans* was 135.25 million years. It was suggested that *C. palmioleophila* originated earlier and belonged to an ancient species of yeast (Figure 5C).

Secondly, this study included *C. palmioleophila* series isolates from the same patient (the isolation time-span was 4 months) (Table 1). The five isolated strains were then compared with the original *C. palmioleophila* 07202534 isolate by WGS. We found that the number of SNPs, indels, and CNVs among the strains was very small, and only one indel site involved an exon (Supplementary Tables S2–S4). This indicated the stability in *C. palmioleophila* genome, which suggested a slow evolution.

Thirdly, by screening ITS gene sequences published on NCBI, more than 30 *C. palmioleophila* strains were compared and analyzed. The results showed that *C. palmioleophila* isolated from different regions at different times had high level of homology. This information also suggests that *C. palmioleophila* evolved slowly.

There was one study found that *C. palmioleophila* has a wide ecological niche, which included marine and soil environments, plants (e.g., crops), and animals (e.g., turtles) (Jackson et al., 2019). These findings are in agreement with another study, which supports the notion that *C. palmioleophila* originated from non-human sources (Corte et al., 2015). As the rapid emergence of *C. auris*, climatic and large-scale environmental changes have encouraged yeast, typically present in the natural environment, to cause more human diseases (Jackson et al., 2019). More worryingly, *C. palmioleophila* (Figure 4) was isolated from cadmium-contaminated paddy soil (MN513331) and wastewater (JN003573). These results suggest that environmental pollution may be one of the important factors that promote *C. palmioleophila* to cause animal and human infection. Thus, *C. palmioleophila*, an ancient and slowly evolving rare fungus, which has been living in natural habitats far from its human hosts, has caused frequent human infections in recent years. The emergence or prevalence of *C. palmioleophila* may be just the tip of the iceberg, warning us that we may face increasing infections with such “dormant” pathogens in the future.

It is also important to note that the full extent of clinical infections caused by *C. palmioleophila* might be underestimated because *C. palmioleophila* can be easily misidentified as *C. famata* (Desnos-Ollivier et al., 2008), *C. albicans* (Casagrande Pierantoni et al., 2020), or *C. guilliermondii* (Jensen and Arendrup, 2011). At present, conventional techniques such as mass spectrometry cannot be used for accurately identifying *C. palmioleophila*. Therefore, clinical laboratories should upgrade their screening methods and performed ITS sequencing or other molecular techniques to improve the identification of *C. palmioleophila* and other similar pathogens (Mota et al., 2012; Eddouzi et al., 2013; Feng et al., 2014; Stavrou et al., 2019).

In conclusion, in this study we reported a case of infection with *C. palmioleophila*, a rare and slowly evolving fungus. In addition we provided a detailed analysis of the microbial and genetic evolutionary characteristics of *C. palmioleophila*, which will help to improve the identification of rare pathogenic fungi and enrich our knowledge of pathogenic fungal species. Future studies should aim to further explore the genetic evolution and the pathogenesis and drug-resistant characteristics of *C. palmioleophila.*

## Data availability statement

The datasets presented in this study can be found in online repositories. The names of the repository/repositories and accession number(s) can be found in the article/Supplementary material.

## Ethics statement

The studies involving human participants were reviewed and approved by the Ethics Review Committee (ERC) of the First Hospital of China Medical University (ERC number: 2019-53-2). Written informed consent for participation was not required for this study in accordance with the national legislation and the institutional requirements.

## Author contributions

NW, YW, YC, ZR, CR, MY, NJ, YJ, and HL made substantial contributions to conception and design, acquisition of data, analysis and interpretation of data. JZ and JC involved in drafting the manuscript. ST have given final approval of the version to be published. All authors contributed to the article and approved the submitted version.

## Funding

This work was supported by the National Key Research and Development Program of China (2021YFC2300400). This work was supported by the National Key Specialist Construction Project for Clinical Laboratory Medicine, Laboratory medicine innovation unit (2019RU017), Chinese Academy of Medical Sciences.

## Conflict of interest

The authors declare that the research was conducted in the absence of any commercial or financial relationships that could be construed as a potential conflict of interest.

## Publisher’s note

All claims expressed in this article are solely those of the authors and do not necessarily represent those of their affiliated organizations, or those of the publisher, the editors and the reviewers. Any product that may be evaluated in this article, or claim that may be made by its manufacturer, is not guaranteed or endorsed by the publisher.

## Footnotes

^1^ http://www.ebi.ac.uk/about/vertebrate-genomics/software/

^2^ http://www.psc.edu/index.php/user-resources/software/picard

^3^ https://www.broadinstitute.org/gatk/download/

^4^ http://mafft.cbrc.jp/alignment/software/

^5^ http://molevol.cmima.csic.es/castresana/Gblocks.html

^6^ http://www.microbesonline.org/fasttree/

^7^ http://www.timetree.org/
