# Supplementary material for: The first rare case of Candida palmioleophila infection reported in China and its genomic evolution in a human host environment
Source: Front Microbiol. 2023 Jul 26;14:1165721. doi: 10.3389/fmicb.2023.1165721 (PMC10469324; doi:10.3389/fmicb.2023.1165721)
Supplement: Supplementary file 1 [file Table_1.DOCX]

Table 1. Characterization and susceptibility testing of the six *Candida palmioleophila* isolates

| Date | Isolate | Source | Colony count cfu/mL | MIC (μg/mL) | | | | | | |
| --- | --- | --- | --- | --- | --- | --- | --- | --- | --- | --- |
|  |  |  |  | FLU | IZ | VOR | AMB | 5-FC | CAS | MF |
| 2020-06-09 | 07202534 | urine | 1x10~5 | 32 | 1 | 1 | 1 | <0.06 | 0.06 | 0.12 |
| 2020-07-13 | 08201515 | urine | 1x10~6 | 32 | 2 | 1 | 1 | <0.06 | 0.06 | 0.12 |
| 2020-07-27 | 08201684 | urine | 1x10~4 | 32 | 4 | 2 | 1 | <0.06 | 0.06 | 0.06 |
| 2020-08-06 | 08201782 | urine | 1x10~5 | 32 | 2 | 1 | 1 | <0.06 | 0.06 | 0.12 |
| 2020-08-18 | 07204494 | urine | 1x10~5 | 32 | 4 | 1 | 1 | <0.06 | 0.12 | 0.06 |
| 2020-10-12 | 07206383 | catheter | 200 | 64 | 1 | 1 | 1 | <0.06 | 0.06 | 0.06 |

*AMB: amphotericin B; 5-FC: flucytosine; FLU: fluconazole; IZ: itraconazole; VOR: voriconazole; CAS: caspofungin; MF: micafungin
