# Supplementary material for: The first rare case of Candida palmioleophila infection reported in China and its genomic evolution in a human host environment
Source: Front Microbiol. 2023 Jul 26;14:1165721. doi: 10.3389/fmicb.2023.1165721 (PMC10469324; doi:10.3389/fmicb.2023.1165721)
Supplement: Supplementary file 2 [file Table_2.DOCX]

Table 2. Genomic assembly and functional annotation of the *Candida palmioleophila* genome

| Item | Value | |  | Item | Count | Percentage(%) |
| --- | --- | --- | --- | --- | --- | --- |
|  | This study | Other study^*^ |  |  |  |  |
| Total length (bp) | 12,762,530 | 12,588,764 |  | NR | 4,343 | 98.41 |
| Max length(bp) | 2,999,745 |  |  | EggNOG | 4,213 | 95.47 |
| GC content (%) | 40.17 | 39.96 |  | KEGG | 2,978 | 67.48 |
| Gene number | 4413 | 5332 |  | SwissProt | 3,968 | 89.92 |
| Total gene number (bp) | 6,821,815 |  |  | GO | 3,605 | 81.69 |
| Gene/Genome (%) | 53.45 |  |  | P450 | 4,314 | 97.76 |
| Contigs | 10 |  |  | TCDB | 980 | 22.21 |
| Scaffolds | 10 | 196 |  | Pfam | 3,779 | 85.63 |
| Contigs N50 | 2004991 |  |  | PHI | 1, 249 | 28.30 |
| Scaffolds N50 | 2004991 | 2030000 |  |  |  |  |
| Contigs N90 | 658531 |  |  |  |  |  |
| Scaffolds N90 | 658531 | 550000 |  |  |  |  |

Note: *other study by Lavergne RA et al.^5^
