# Supplementary material for: The first rare case of Candida palmioleophila infection reported in China and its genomic evolution in a human host environment
Source: Front Microbiol. 2023 Jul 26;14:1165721. doi: 10.3389/fmicb.2023.1165721 (PMC10469324; doi:10.3389/fmicb.2023.1165721)
Supplement: Supplementary file 3 [file Table_3.DOCX]

Supplement figure C*. palmioleophila* Genome Annotation

Supplement figure1 Functional annotation of *C.-palmioleophila*-gene-encoding proteins using the EggNOg database

The EggNOg database showed that most of the predicted *C. palmioleophila* genes were functionally associated with “Function unknown” (981 genes), “Translation, ribosomal structure and biogenesis”, and “Post-translational modification, protein turnover, chaperones”, “Intracellular trafficking, secretion, and vesicular”, “Transcription”, “Carbohydrate transport and metabolism” in that order. The enrichment of functions relating to post-translational events and carbohydrate metabolism suggested that the predicted genes were involved in the regulation of protein bioactivity and energy conversion efficiency (Supplementary Figure 1).


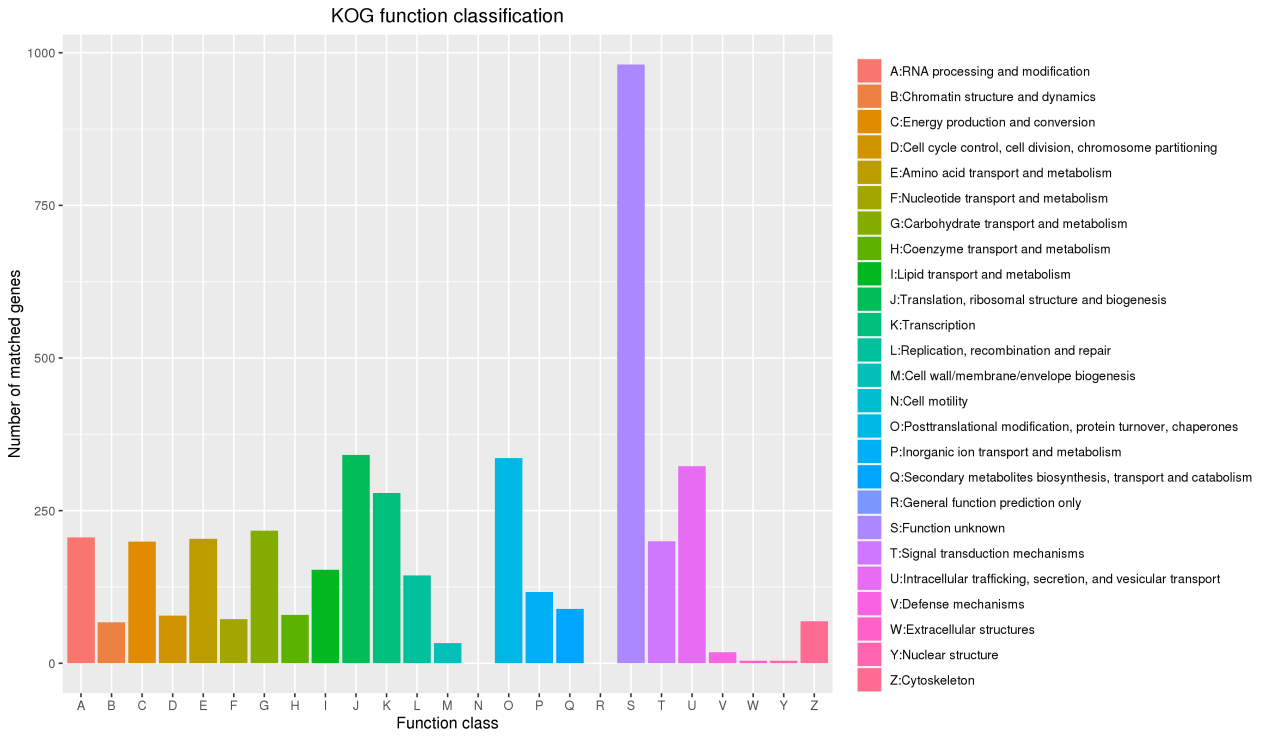


Supplement figure1 Functional annotation of *C.-palmioleophila*-gene-encoding proteins using the EggNOg database

Supplement figure2 Functional annotation of *C.-palmioleophila*-gene-encoding proteins using KEGG

The KEGG functional classification revealed that the predicted *C. palmioleophila* genes were associated with genetic information processing (2,154 genes), signaling and cellular processing (472 genes), signal transduction (481 genes), and carbohydrate metabolism (307 genes). The enrichment of functins relating to the processing of genetic information and protein signaling suggest that these genes may faciliate efficient information exchange and secondary metabolism (Supplementary Figure 2).


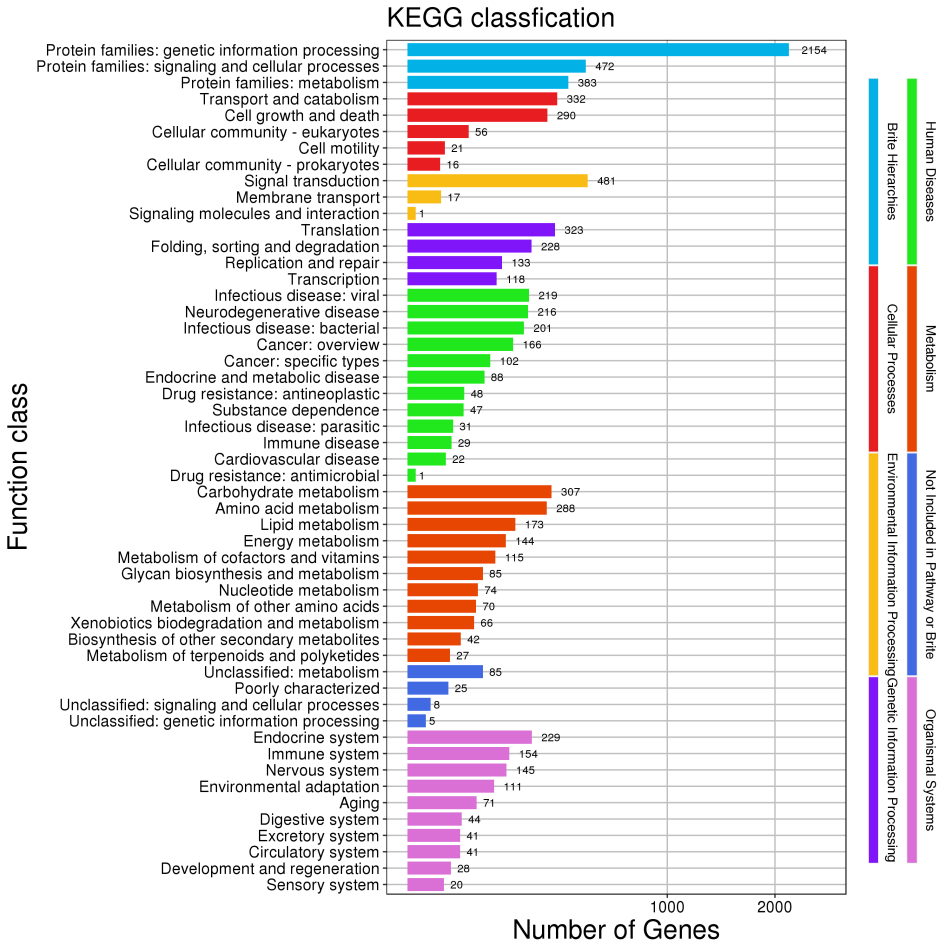


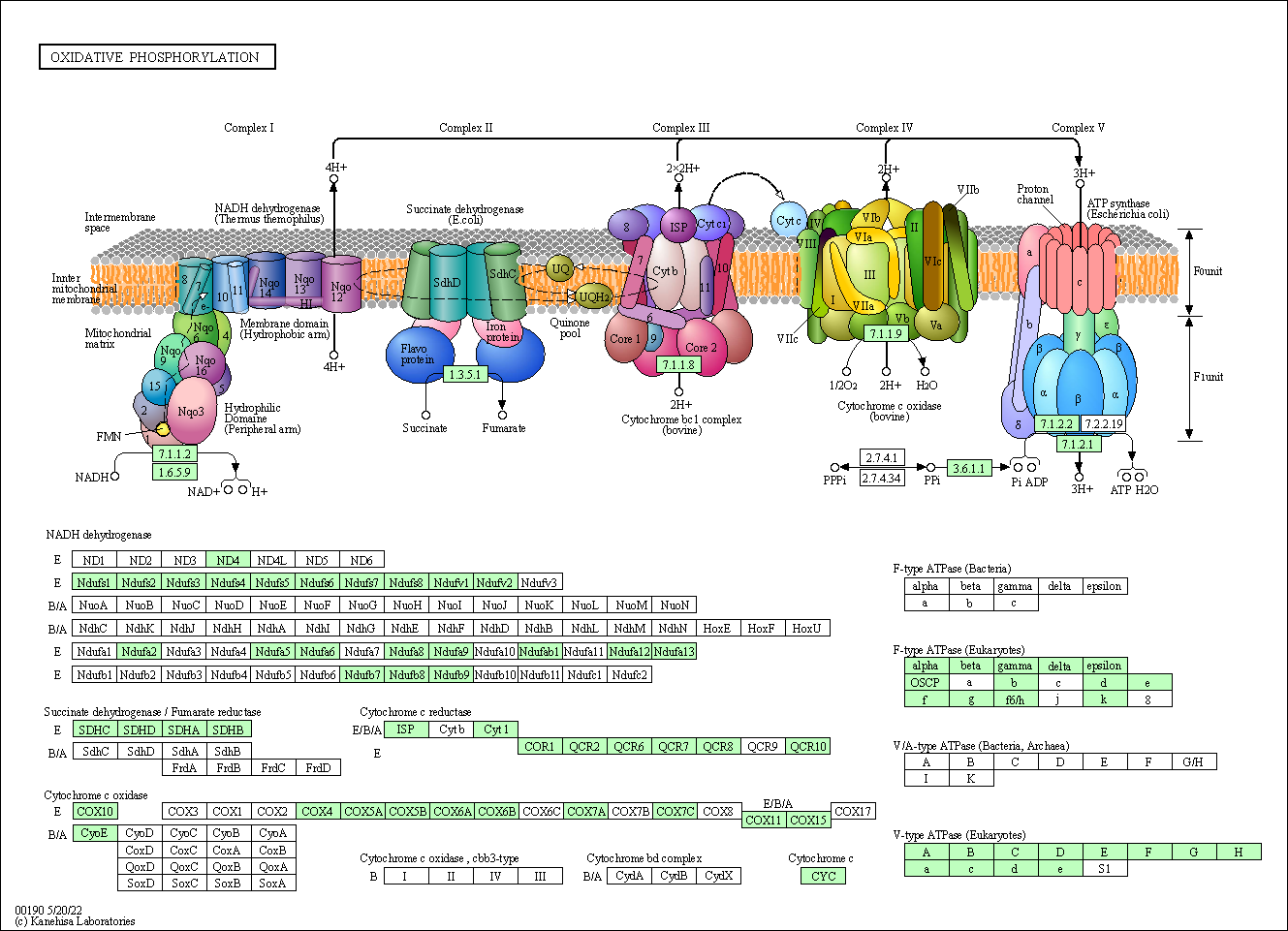


Supplement figure2 Functional annotation of *C.-palmioleophila*-gene-encoding proteins using KEGG

Supplement figure3 Functional annotation of *C.-palmioleophila*-gene-encoding proteins using the GO database

GO annotation of the predicted *C. palmioleophila* genes yielded 3,271 genes that were enriched terms associated with “biological processes”. Further annotation revealed that the genes were associated with molecular function (2,821 genes) from “molecular function”, and cell (2,195 genes), intracellular (2,119 genes), organelle (1,662 genes), cellular component (1,604 genes), and cytoplasm (1,475 genes) from “cellular component”. Thus, many of these metabolic genes might be involved in signal transduction (Supplementary Figure 3).


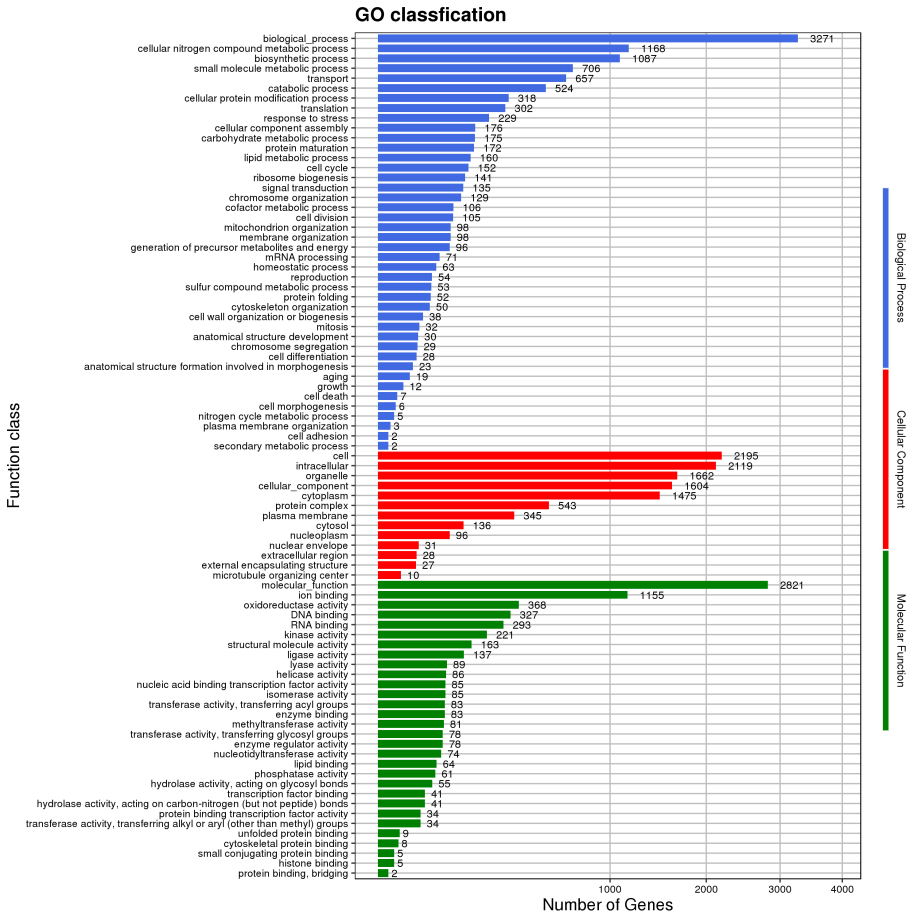


Supplement figure3 Functional annotation of *C.-palmioleophila*-gene-encoding proteins using the GO database

Supplement figure 4 Functional annotation of *C.-palmioleophila*-gene-encoding proteins using the PHI database

The amino acid sequences of *C. palmioleophila* were aligned to obtain annotated results from the PHI database. We found that the *C. palmioleophila* genome contained genes associated with reduced virulence (705 genes), unaffected pathogenicity (388 genes), loss of pathogenicity (159 genes), lethality (87 genes), increased virulence (hypervirulence; 45 genes), effector (plant avirulence determinant; 4 genes), and resistance to chemicals (3 genes). The results showed that the main annotated genes were implicated in reduced virulence and unaffected pathogenicity, indicating that *C. palmioleophila* was a mildly pathogenic strain (Supplementary Figure 4).


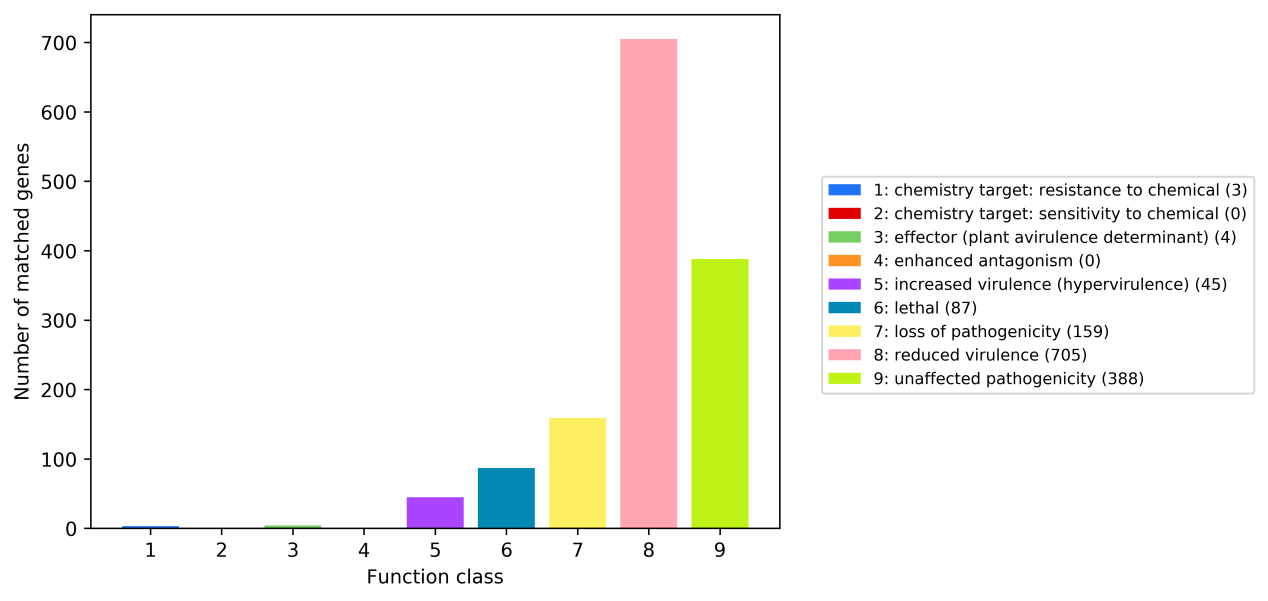


Supplement figure 4 Functional annotation of *C.-palmioleophila*-gene-encoding proteins using the PHI database

Supplement figure 5 Functional annotation of *C.-palmioleophila*-gene-encoding proteins using the CAZy database

The CAZy analysis revealed 143 genes encoding carbohydrate-active enzymes in *C. palmioleophila* genome, including 56 glycoside hydrolases, 56 glycosyl transferases, 15 carbohydrate esterases, 13 auxiliary activities, and 3 carbohydrate-binding modules. These findings suggest that *C. palmioleophila* may be capable of increasing its energy supply and decomposing complex carbohydrates (Supplementary Figure 5).


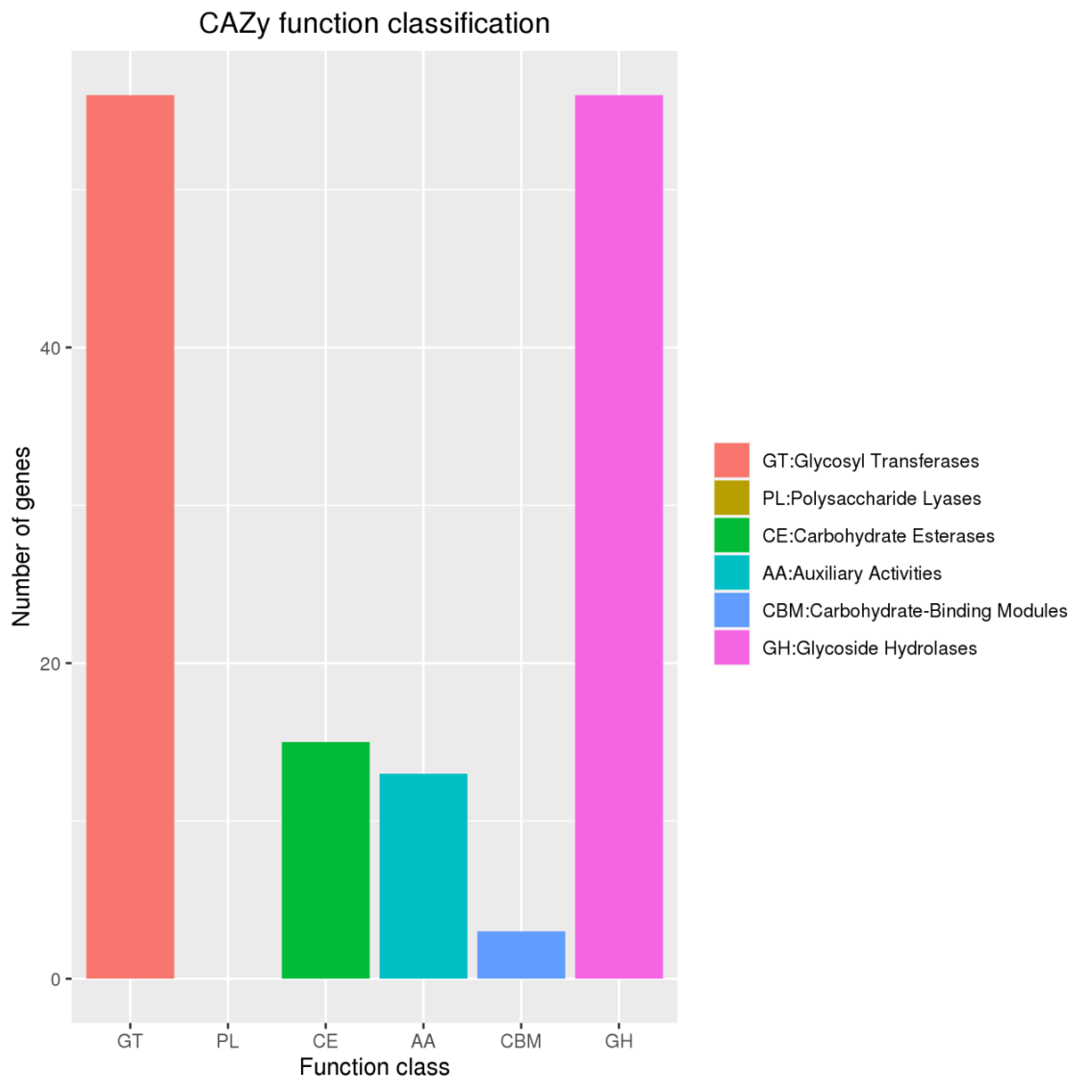

Supplement figure 5 Functional annotation of *C.-palmioleophila*-gene-encoding proteins using the CAZy database

Supplement figure 6 Functional annotation of *C.palmioleophila* expanded and contracted genes encoding proteins using KEGG

Annotation of expanded genes in KEGG database involved Metabolism, Brite Hierarchies, Human Diseases and Environmental Information Processing in turn.

contracted gene annotation in KEGG database involves Human Diseases, Brite Hierarchies, Organismal Systems and Metabolism in turn. The increased function of genes in Metabolism and the decreased function of genes in Human Diseases suggest that *C.palmioleophila* has enhanced its ability to adapt to the environment in the process of evolution (Supplementary Figure 6).


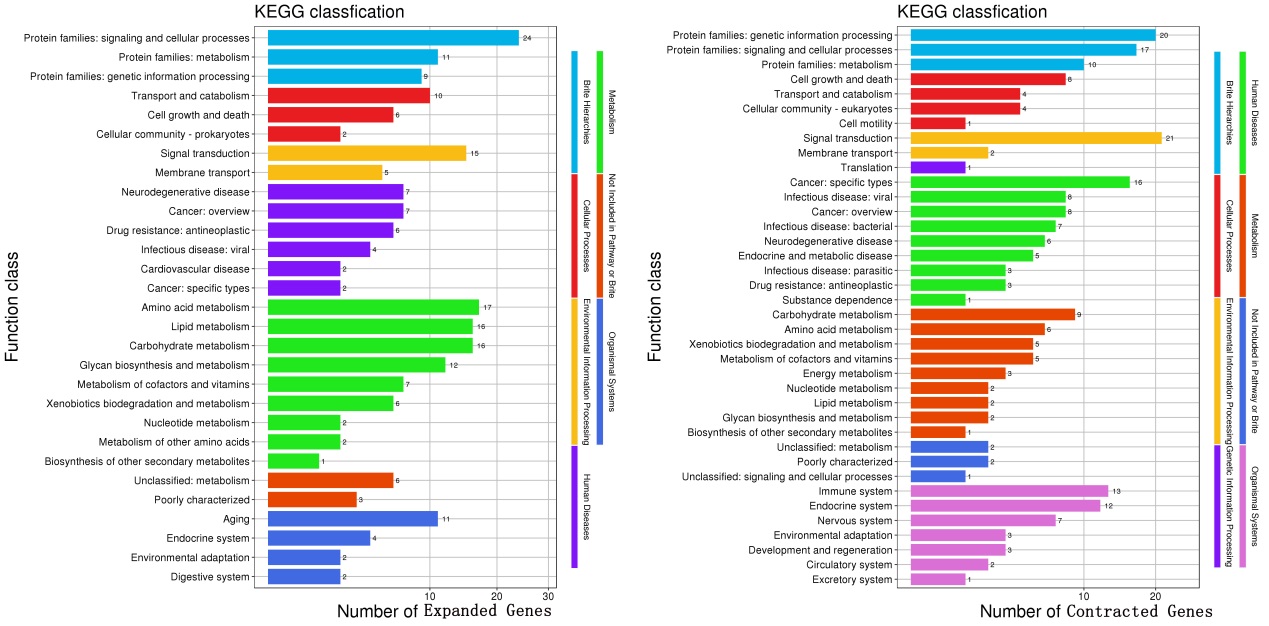


Supplement figure 6 Functional annotation of *C.palmioleophila* expanded and contracted genes encoding proteins using KEGG

Supplement figure 7 Functional annotation of *C.palmioleophila* expanded and contracted genes encoding proteins using GO database

No matter expanded genes or contracted genes are enriched into three categories when using GO database for functional annotation: "biological processes", "molecular function", and cellular component. Thus, many of these metabolic genes might be involved in signal transduction (Supplementary Figure 7).


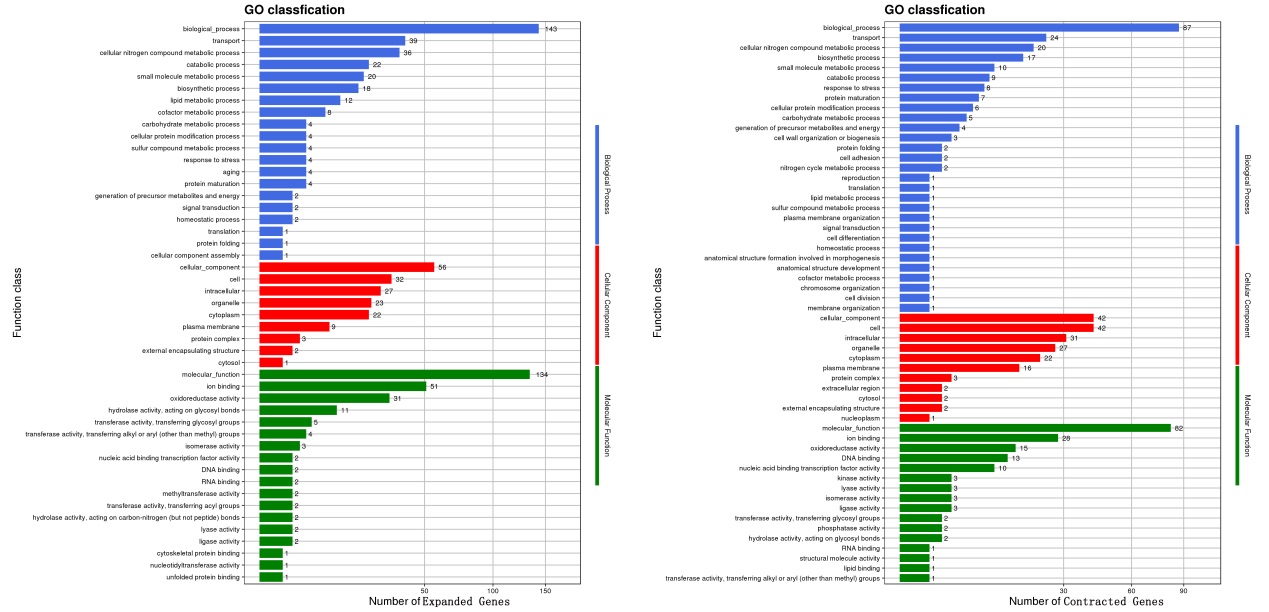


Supplement figure7 Functional annotation of *C.palmioleophila* expanded and contracted genes encoding proteins using GO database
