# Supplementary material for: The first rare case of Candida palmioleophila infection reported in China and its genomic evolution in a human host environment
Source: Front Microbiol. 2023 Jul 26;14:1165721. doi: 10.3389/fmicb.2023.1165721 (PMC10469324; doi:10.3389/fmicb.2023.1165721)
Supplement: Supplementary file 8 [file Table_8.DOCX]

**Supplementary Table 5. Genome accession numbers of related fungal species used in the genomic comparative analysis**

| **Species** | **The accession number of the genome** |
| --- | --- |
| *Saccharomyces cerevisiae* | [GCA_000146045.2](https://www.ncbi.nlm.nih.gov/assembly/GCA_000146045.2) |
| *Candida albicans* | [GCA_000182965.3](https://www.ncbi.nlm.nih.gov/assembly/GCA_000182965.3) |
| *Debaryomyces hansenii* | [GCA_000006445.2](https://www.ncbi.nlm.nih.gov/assembly/GCA_000006445.2) |
| *Schizosaccharomyces pombe* | [GCA_000002945.2](https://www.ncbi.nlm.nih.gov/assembly/GCA_000002945.2) |
| *Scheffersomyces stipitis* | [GCA_000209165.1](https://www.ncbi.nlm.nih.gov/assembly/GCA_000209165.1) |
| *Candida tropicalis* | [GCA_000006335.3](https://www.ncbi.nlm.nih.gov/assembly/GCA_000006335.3) |
| *Candida glabrata* | [GCA_020450195.1](https://www.ncbi.nlm.nih.gov/assembly/GCA_020450195.1) |
| *Yarrowia lipolytica* | [GCA_014490615.1](https://www.ncbi.nlm.nih.gov/assembly/GCA_014490615.1) |
| *Candida maltosa* | GCA_000344705.1 |
| *Meyerozyma guilliermondii* | [GCA_000149425.1](https://www.ncbi.nlm.nih.gov/assembly/GCA_000149425.1) |
| *Candida parapsilosis* | [GCA_000182765.2](https://www.ncbi.nlm.nih.gov/assembly/GCA_000182765.2) |
| *Clavispora lusitaniae* | [GCA_009498055.1](https://www.ncbi.nlm.nih.gov/assembly/GCA_009498055.1) |
| *Lodderomyces elongisporus* | [GCA_000149685.1](https://www.ncbi.nlm.nih.gov/assembly/GCA_000149685.1) |
| *Candida dubliniensis* | [GCA_000026945.1](https://www.ncbi.nlm.nih.gov/assembly/GCA_000026945.1) |
| *Cyberlindnera jadinii* | [GCA_001661405.1](https://www.ncbi.nlm.nih.gov/assembly/GCA_001661405.1) |
| *Candida auris* | [GCA_003013715.2](https://www.ncbi.nlm.nih.gov/assembly/GCA_003013715.2) |
| *Candida intermedia* | *GCA_900106125.1* |
| *Diutina rugosa* | *GCA_008704595.1* |
| *Candida metapsilosis* | GCA_017655625.1 |
| *Candida orthopsilosis Co 90-125* | GCA_000315875.1 |
| *Candida viswanathii* | GCA_003327735.1 |
| *Debaryomyces fabryi* | *GCA_001447935.2* |
| *Suhomyces tanzawaensis NRRL Y-17324* | GCA_001661415.1 |
